# The effect of rugby training on indirect markers of gut permeability and gut damage in academy level rugby players

**DOI:** 10.1007/s00421-022-05027-w

**Published:** 2022-09-02

**Authors:** Sarah Chantler, Alex Griffiths, Padraic Phibbs, Gregory Roe, Carlos Ramírez-López, Glen Davison, Ben Jones, Kevin Deighton

**Affiliations:** 1 Carnegie Applied Rugby Research (CARR) Centre, Carnegie School of Sport, Leeds Beckett University, Leeds, UK; 2 Yorkshire Carnegie Rugby Union Club, Leeds, UK; 3 School of Clinical and Applied Sciences, Leeds Beckett University, Leeds, UK; 4 Centre of Excellence, Leinster Rugby, Dublin, Ireland; 5 Bath Rugby Union Club, Bath, UK; 6 Scottish Rugby Union, Edinburgh, UK; 7 School of Sport and Exercise Sciences, Division of Natural Sciences, University of Kent, Canterbury, UK; 8 School of Science and Technology, University of New England, Armidale, NSW Australia; 9 Division of Exercise Science and Sports Medicine, Department of Human Biology, Faculty of Health Sciences, the University of Cape Town and the Sports Science Institute of South Africa, Cape Town, South Africa; 10 Leeds Rhinos Rugby League Club, Leeds, UK; 11 England Performance Unit, Rugby Football League, Leeds, UK; 12 Delta Hat Limited, Tamworth Road, Nottingham, UK

**Keywords:** Rugby, Gastrointestinal, Health, Permeability, Exercise

## Abstract

**Purpose:**

To assess indirect markers of intestinal endothelial cell damage and permeability in academy rugby players in response to rugby training at the beginning and end of preseason.

**Methods:**

Blood and urinary measures (intestinal fatty acid binding protein and lactulose:rhamnose) as measures of gastrointestinal cell damage and permeability were taken at rest and after a standardised collision-based rugby training session in 19 elite male academy rugby players (age: 20 ± 1 years, backs: 89.3 ± 8.4 kg; forwards: 111.8 ± 7.6 kg) at the start of preseason. A subsample (*n* = 5) repeated the protocol after six weeks of preseason training. Gastrointestinal symptoms (GIS; range of thirteen standard symptoms), aerobic capacity (30–15 intermittent fitness test), and strength (1 repetition maximum) were also measured.

**Results:**

Following the rugby training session at the start of preseason, there was an increase (median; interquartile range) in intestinal fatty acid binding protein (2140; 1260–2730 to 3245; 1985–5143 pg/ml, *p* = 0.003) and lactulose:rhamnose (0.31; 0.26–0.34 to 0.97; 0.82–1.07, *p* < 0.001). After six weeks of preseason training players physical qualities improved, and the same trends in blood and urinary measures were observed within the subsample. Overall, the frequency and severity of GIS were low and not correlated to markers of endothelial damage.

**Conclusions:**

Rugby training resulted in increased intestinal endothelial cell damage and permeability compared to rest. A similar magnitude of effect was observed after six weeks of pre-season training. This was not related to the experience of GIS.

## Introduction

Acute exercise contributes to a significant increase in markers of endothelial cell damage and gut permeability (Costa et al. 2017; Chantler et al. 2020). This initial phase of exercise associated gastrointestinal syndrome may include bacterial translocation and an elevated inflammatory response (Li et al. 2013; Shing et al. 2014; Costa et al. 2017). Studies have found elevated cell permeability, endotoxin levels or inflammatory markers with a concomitant increase in gastrointestinal symptoms (GIS) (Li et al. 2013; Shing et al. 2014; Stuempfle et al. 2016; Islam et al. 2017). Exercise-associated GIS can directly affect performance (Hoffman and Fogard 2011), but endothelial cell damage may also have downstream effects on recovery and nutrient absorption (van Wijck et al. 2013a).

Different mechanisms for exercise associated GIS have been investigated in endurance exercise, often using steady state protocols (Chantler et al. 2020). This limits the application towards high intensity intermittent or team sports (e.g. soccer, rugby), especially as many team sports include multiple modalities within a training program (e.g. conditioning, skills, resistance training). Although studies are limited, resistance exercise as well as repeated high intensity sprints have been found to increase markers of gut endothelial disruption and GIS similar to endurance exercise (van Wijck et al. 2013a; Pugh et al. 2017b). Team sports may combine multiple elements described above and may therefore be susceptible to elevated endothelial cell damage and permeability, as the starting point of gastrointestinal cell damage. However, there is currently no research investigating the gastrointestinal cell response to exercise in team sports.

Rugby consists of repeated bouts of high intensity running interspersed with collisions (Reardon et al. 2017). Rugby players will often include both rugby-specific training, conditioning and resistance training sessions as part of their training programs (Bradley et al. 2015). Collisions increase energy requirements and markers of muscle damage compared to running, which adds another dimension compared to other team sports (Roe et al. 2017; Costello et al. 2019). Multiple training sessions per day and high body masses suggests there should be a focus on recovery (Bradley et al. 2015). Gastrointestinal endothelial cell damage has been correlated to lower levels of absorbed amino acids post-exercise (van Wijck et al. 2013a), but with no effect on absorption of milk-based carbohydrate (Costa et al. 2020b). These studies are in acute response to exercise, and hypothetically, repeated bouts of high intensity exercise may not allow for complete endothelial recovery as endothelial cell maturation takes place over days rather than hours (Marshman et al. 2002; Van Houten et al. 2015). While data suggest that biomarkers of endothelial cell damage return to normal within 1–2 h post-exercise (van Wijck et al. 2011), overall gastrointestinal cell recovery, immuno-tolerance, or nutrient absorption may be negatively impacted by rugby, indicated by changes in gut endothelial integrity in previous research (van Wijck et al. 2013b; Li et al. 2013). As such, the impact of training on endothelial response as part of the differentiating acute and chronic responses warrants investigation.

Therefore, the aim of this study was to assess biomarkers (i.e. intestinal fatty acid binding protein and dual sugar absorption test) of gut endothelial cell damage and permeability in response to (part 1) a single standardised collision-based rugby training session at the start of preseason, and to (part 2) repeat the protocol after six weeks of training in elite academy level rugby union players. An additional aim was to assess the experience of GIS at rest and during rugby training and match play that may extend from these findings.

## Methods

### Participants

Twenty-one male rugby players from two regional Premiership rugby union academies (age 20 ± 1 years) were invited as a convenient sample to participate in the study during their preseason. All participants were healthy and were declared injury free and fit to train by their medical teams. Participants declared they were free from any diagnosed gastrointestinal disorders (e.g. Crohn’s, Ulcerative colitis, irritable bowel disorder). All players from the academies agreed to participate and informed consent was obtained from all participants prior to testing. Ethics was approved by University Research Ethics committee.

Twenty-one participants completed the initial measures (i.e., L:R; i-FABP) at rest. Two participants who completed these measures were unable to complete the training trial due to onset of illness and were subsequently excluded. Nineteen players completed part 1 (i.e. Rest and Training trials) and were included in the subsequent analysis. After six weeks of preseason training (during June and July), players from one academy were retested (part 2) to examine any influence of chronic training on gastrointestinal endothelial response. Due to injuries and illness occurring during preseason, the original subsample from this academy was reduced from ten to five players.

### Study design

**Fig. 1:**
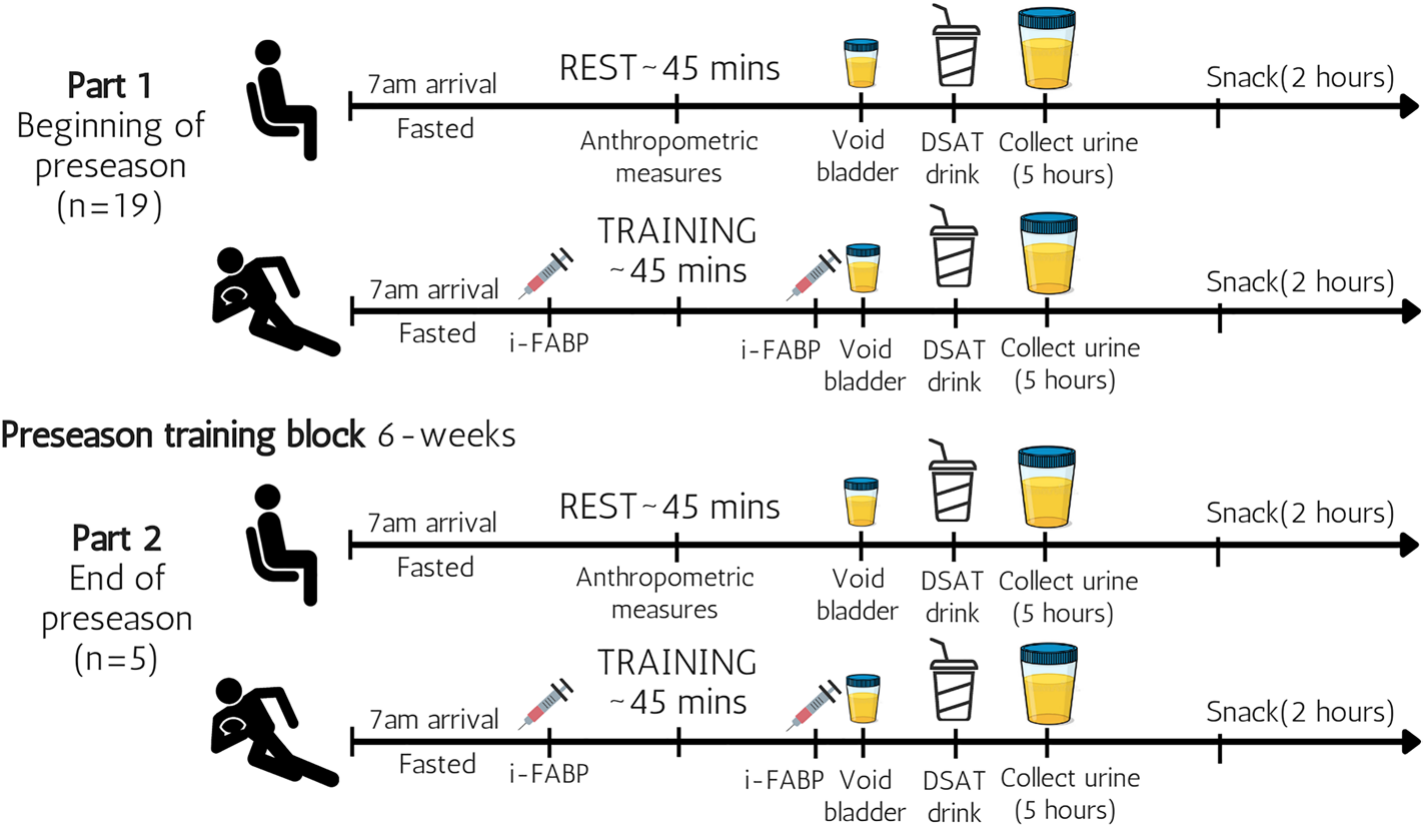
Schematic diagram of the Rest and Training protocol at the beginning (part 1) and end of preseason (part 2) including blood (i-FABP) and urine (Lactulose/Rhamnose) measures, Snack: was a high protein egg-based snack as described in methods

In a within-subjects study design, participants completed one trial at rest (Rest) and one rugby training session (Training) (Fig. 1). Initial testing was performed at the beginning of the pre-season training block after the off-season (4-week duration). This period was selected to compare with the end of pre-season after six weeks of training (part 2). Participants were asked to avoid caffeine, alcohol, non-steroidal anti-inflammatory drugs (NSAIDs), spicy food and strenuous exercise for the 24 h prior to each testing due to their impact on endothelial cells (Marchbank et al. 2011; Van Wijck et al. 2012). The participants arrived at 7am after an overnight fast. Participants were asked to have their last meal prior to 10 pm. Water was permitted ad libitum. For the Rest trial, participants rested for ~ 45 min to simulate the timing of the Training trial. The Training was performed the day after a full rest day in the first week of pre-season training (Academy one ~ 2 days and Academy two ~ 3 days after Rest testing, respectively). Due to the invasive nature of taking blood, venous blood sampling was limited to the Training trial (pre and post) to reduce the burden on the participants.

### Training trial

**Fig. 2:**
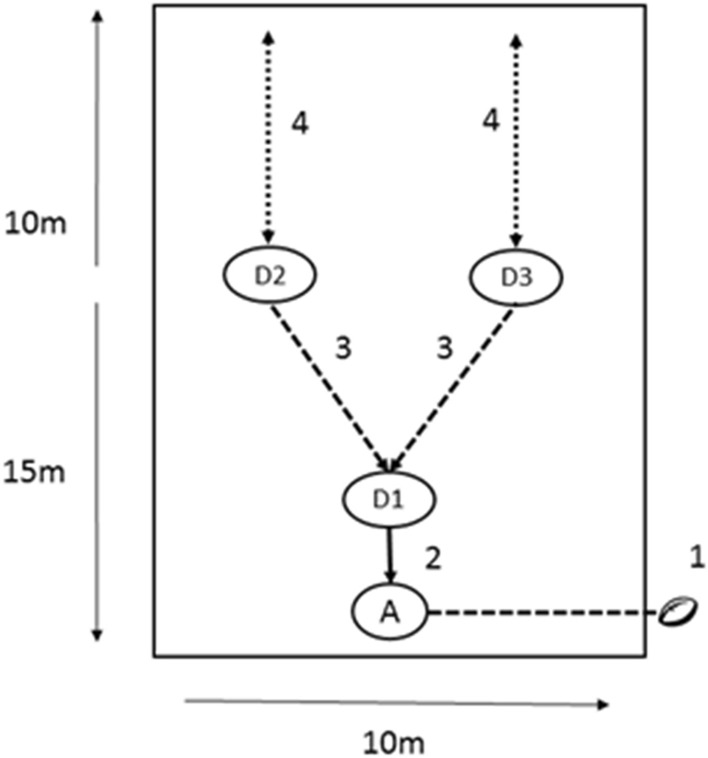
Schematic representation of the rugby training session; A, attacker; D1, D2, D3, defenders. The first collision is between A and D1 (2), with support from D2 and D3 (3), after which, D2/D3 complete a shuttle run (4)

The high intensity collision-based training session was designed to combine elements of running fitness, basic skills and conditioning for rugby. All players were familiar with the style of drill and conditioning as part of normal training. The trial was completed outside in temperate conditions (temperature 21 and 16 °C, 72% and 66% relative humidity in two venues, respectively). After a standard warm up with the rugby coaching staff, the participants completed 3 × 8-min blocks of a rugby-specific conditioning exercise with two minutes rest between (Fig. 2). The training protocol was based upon a 4-person rugby-based situational practice, with an emphasis on evasion skills, collision, and within-contact exertion (collision-based conditioning). Positional groups (forwards and backs) were separated for the protocol, to ensure no mismatches in the collisions.

Each group of four participants rotated through four different roles, one attacking (A1), one defending (D1), and two as support defenders (D2 and D3). Each player completed twelve timed contacts per block (five seconds each; three as an attacker, nine in the various defending positions) and completed six 20-s shuttle runs at 95% of their individual 30–15 intermittent fitness test (IFT) score (Buchheit 2008) while positioned as a support defender (D2 and D3) within the eight-minute block. There was no rest during the 8-min block. This session was designed to be specific to the physiological demands of rugby conditioning while controlling for the number and style of collisions to reduce the risk of injuries associated with full-contact training (Cousins et al. 2019). GPS units (Catapult S5) and heart rate monitors (Polar) were used to monitor the players physically during the session.

### Urinary measures

Intestinal permeability was measured at the end of Rest and immediately after Training via the ingestion of a dual saccharide drink (dual saccharide absorption test [DSAT]) as per a previous protocol (Playford et al. 2001; Marchbank et al. 2011; Davison et al. 2016). The players were asked to void their bladders after the rest period, or directly after training, prior to consuming the DSAT. Water intake was limited for the first hour after which they were encouraged to drink water ad libitum. Participants collected their urine in individual containers for the subsequent 5 h.

An egg-based snack (~ 20 g protein) was given 2 h after consuming the DSAT drink. The recipe and the timing were to ensure that there was no interference with the saccharide probes (Lactulose, Rhamnose) by any additional form of carbohydrate during the DSAT transit through the gut (Van Nieuwenhoven et al. 2000). Urine production and water intake was tracked over the 5 h via the use of a digital scale. After the total volume was recorded, a pooled sample was centrifuged to remove gross debris and the supernatant was frozen at  – 20 °C. On further analysis, the sugars were separated by high-performance liquid chromatography and quantified using a pulsed amphometric detector (Marchbank et al. 2011). The sugars are oxidised on the gold electrode at the working potential (0.05 V) and the current measured reflects the amount of sugar in the sample. The sugars (Lactulose and Rhamnose) were expressed as a ratio.

### Blood measures

Intestinal fatty acid binding protein (i-FABP) was used to assess intestinal endothelial cell damage (Derikx et al. 2017). A 5 ml blood sample was collected into heparinised tubes directly pre and post training, via the anti-cubital vein. After a small subsample was removed for haemoglobin and haematocrit measures, the remaining sample was centrifuged immediately at 3000 rpm for 10 min at 4 °C. The plasma fraction was removed and stored at  – 80 °C for later analysis. I-FABP was analysed using a commercial ELISA kit according to the manufacturer’s instructions (Quantikine®, R&D Systems, Minneapolis, USA).

### Other measures

The participants completed an online 24-h dietary recall for the day prior to both rest and training trials. Participants were asked to match their dietary intake for both to ensure consistent intakes, and the dietary intakes were reviewed prior to the trails by the researchers. All participants matched their dietary intake. An adapted GIS screening questionnaire (adapted from Pugh et al. 2018) was completed at Rest to investigate self-reported GIS at rest and previous rugby training and match play. Thirteen traditional GIS, with the addition of loss of appetite and extreme hunger were included. The scale of symptoms was modified to align with the other wellness monitoring done with the players (McLean et al. 2010). The severity of symptoms was rated on a scale of 0–5, with the descriptions of (0) nothing; (1) just noticeable, not painful; (2) noticeable; (3) quite noticeable, mildly painful or disruptive; (4) painful, quite disruptive, but does not prevent any day-to-day activities; (5) painful, may need medication and disrupts the flow of daily activities. The GIS screening questionnaire was completed online via the participant’s phones for convenience. This has not been previously validated in this population. Anthropometric and body composition measures (stature, body mass and skinfolds) were taken during the resting trial. An International Society for the Advancement of Kinanthropomery (ISAK) Level 1 accredited practitioner took the skinfolds of all the participants. Body mass (kg) and stature (cm) were taken on a Seca scale (model 22,089) and Seca Stadiometer, respectively. To estimate exercise associated fluid loss, body mass was taken before and after the Training trial, and once at the beginning of the Rest trial as per ISAK standards, prior to urination. Haemoglobin and blood haematocrit levels were measured pre and post-exercise to monitory plasma based changes in relation to body weight losses (Dill and Costill 1974). Strength testing (1 repetition maximum) and an estimation of fitness (30–15 test (Buchheit 2008)) were completed at baseline and again after six weeks of preseason.

### End of pre-season measures (part 2)

The full protocol was repeated with a subsample of players (*n* = 5) after six weeks of pre-season training (temperature: 16 °C and 85% humidity).

### Statistical analysis

Statistical analysis was conducted using the Statistical Package for the Social Sciences software programme (SPSS, version 26). Normally distributed data are expressed as mean ± standard deviation (SD), while non-normally distributed data are presented as median (interquartile range). Due to some variables (i.e. L:R, i-FABP) being non-normally distributed, non-parametric tests were used throughout. Wilcoxon signed rank test and Spearman’s correlations were used to establish the impact of exercise and the relationship between biomarkers. *P* < 0.05 was considered statistically significant. Due to the small sample size in part 2 (n = 5), there were no statistics performed during end of preseason testing.

## Results

### Baseline

The physical characteristics of the participants are presented in Table 1. The forwards were significantly heavier with higher total skinfolds (mm) (sum of eight sites) compared to backs (*p* < 0.05).

**Table 1:** Mean ± standard deviation player (anthropometric, strength and fitness) characteristics at the beginning of preseason

	All (*n* = 19)	Forwards (*n* = 9)	Backs (*n* = 10)
Age (years)	20 ± 1	20 ± 1	20 ± 1
Body mass (kg)	100.0 ± 13.9	111.8 ± 7.6	89.3 ± 8.4*
Stature (cm)	184.4 ± 7.3	188.6 ± 7.1	181.1 ± 5.6
Σ SF (mm)	94.6 ± 32.5	119.1 ± 26.8	72.6 ± 18.5*
1RM Back Squat (kg)	163 ± 22	173 ± 28	155 ± 11
1RM Bench Press (kg)	117 ± 17	124 ± 17	111 ± 14
30–15 IFT (stage)	18.6 ± 1.3	18.3 ± 1.5	19.0 ± 1.2

*Kg* kilograms; cm, centimetres, *Ʃ SK* sum of skinfolds (8 sites), *mm* millimetres, *1RM* one repetition maximum, *IFT* intermittent fitness test; km/h. kilometres per hour; *denotes a significant difference between the two positional groups, (*p* < 0.05)

**Fig. 3:**
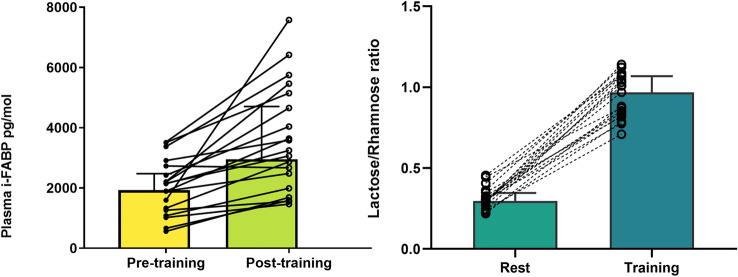
Beginning of preseason (part 1) individual change and group median (IQR) values of (left) plasma i–FABP (pg/ml) pre and post Training (*n* = 19), *p* < 0.001; and individual change and group median (IQR) (right) Lactulose/Rhamnose at Rest and Training trials (*n* = 19), *p* < 0.001

**Fig. 4:**
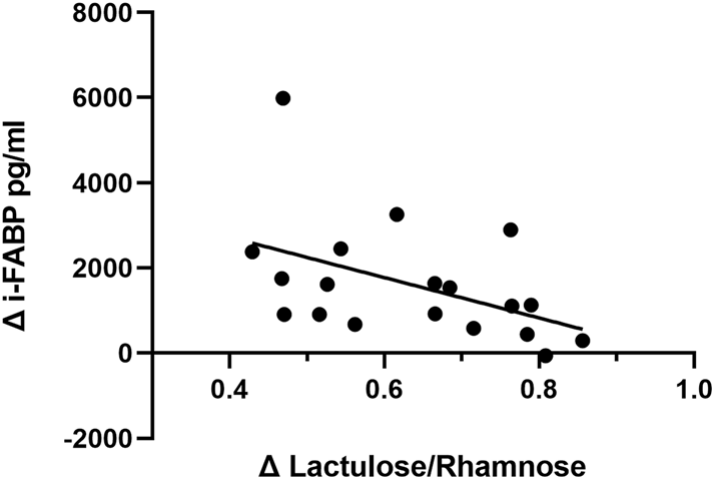
Spearman’s correlation coefficient between absolute change in permeability and cell damage biomarkers (i-FABP and Lactulose/Rhamnose) for individual participants; *r* =  – 0.54, *p* = 0.018

The mean total distance covered for Training was 555 ± 23 m with a mean heart rate of 170 ± 9 beats/min. Training resulted in a significant decrease in body mass compared to pre-training (1.1 ± 0.5 kg; *p* < 0.001). After adjusting for changes in plasma volume, i-FABP levels increased 70% from 2140 (1260–2730) to 3245 (1985–5143) pg/ml (Fig. 3, *p* = 0.003). Urinary lactulose:rhamnose (L:R) increased significantly by 211% between Rest and Training conditions from 0.31 (0.26–0.34) to 0.97 (0.82–1.07) (Fig. 3, *p* < 0.001). Spearman’s correlation coefficient showed a negative correlation between the absolute change in L:R and i-FABP (Fig. 4, *r* =  – 0.58, *p* = 0.018). A narrow range of GIS was reported around Rest and rugby training and match play (Table 2). Burping was the most commonly reported upper GIS, with higher frequencies at rest compared to rugby training or match play. The median severity of symptoms (on a scale of 0–5) was between zero and one (nothing to just noticeable) for all symptoms at both rest and during rugby training or match play.

**Table 2:** Self-reported frequency and severity of gastrointestinal symptoms at rest and during rugby training and match play

Gastrointestinal symptoms	GIS frequency at rest (*n*)	GIS Severity	GIS frequency during rugby (*n*)	GIS Severity
Heartburn	0	1 (0–1.5)	0	0 (0–1)
Burping	9	1 (0.5–1)	4	0 (0–1)
Upper abdominal pain	0	0 (0–1)	0	0 (0–1)
Nausea	0	0 (0–1)	1	0 (0–1)
Vomiting	0	0 (0–1)	0	0 (0–1)
Stomach cramps or gurgling	0	1 (0–1)	1	0 (0–1)
Bloating	3	1 (0–1)	2	1 (0–1)
Lower abdominal pain	0	0 (0–1)	0	0 (0–1)
Flatulence	4	1 (0–1)	3	0 (0–1)
Constipation	0	0 (0–1)	0	0 (0–1)
Diarrhoea	1	0 (0–1)	0	0 (0–1)
Urgent need to defecate	0	0 (0–1)	0	0 (0–1)
Change in stool consistency	4	1 (0–1.5)	2	0 (0–1.5)
Loss of appetite	1	0 (0–1)	3	0 (0–1)
Extreme bouts of hunger	6	1 (0–1)	2	0 (0–1)
Upper GIS total	6	4 (0.5–6.5)	4	1 (0–6)
Lower GIS total	6	4 (1–8)	5	2 (0–7)*
All GIS total	11	7 (2–14.5)	5	3 (0–13)

Data are presented as frequency of the number of players who reported experiencing any symptom once a week or more often; and severity, as median (IQR) rating of discomfort on a scale of 0–5 (*n* = 19); *denotes a difference in severity between rest and rugby, *p* < 0.05

### End of preseason testing

**Fig. 5:**
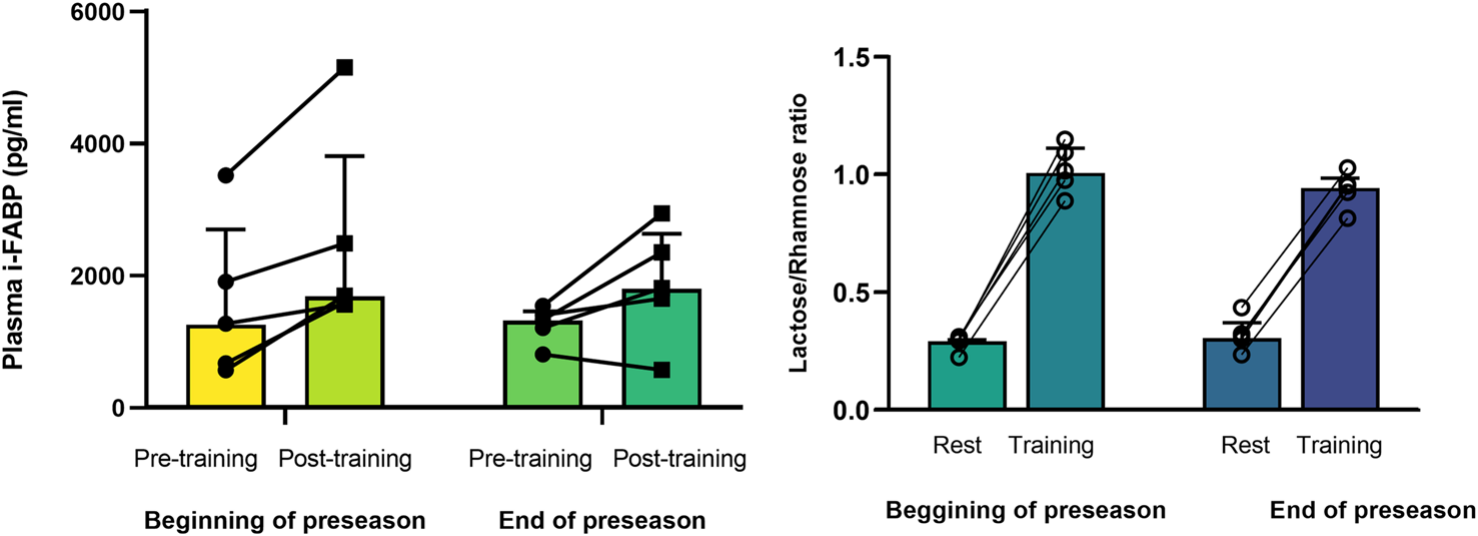
Beginning and end of preseason (part 2) individual change and group median (IQR) values of (left) plasma i–FABP (pg/ml) pre and post Training; and individual change and median (IQR) of (right) Lactulose/Rhamnose at Rest and Training at same time points

At the end of the preseason four backs and one forward repeated the testing (Table 3). Body composition, aerobic capacity (30–15) and strength measures (1RM) improved (Table 3). After repeating the Training (body mass loss during Training trial: 1.0 ± 0.3 kg,) the median change of i-FABP was lower after six weeks (606 [248 – 1019] pg/ml, Fig. 5) compared to at the beginning of preseason (921 [580 – 1130] pg/ml, Fig. 5). The lactulose:rhamnose ratio increased at both time points from 0.29 (0.29–0.29) to 1.01 (0.97–1.99) and 0.31 (0.26–0.37) to 0.94 (0.87–0.99) at the start and end of preseason, respectively but was comparatively lower at the second testing (Fig. 5).

**Table 3:** Player (anthropometric, strength and fitness) characteristics of the subsample at the beginning and end of preseason

	All	Scrumhalf	Centre	Centre	Flyhalf	Hooker
Beginning preseason body mass (kg)	93.5 ± 9.1	83.2	86.5	102.5	89.3	106.3
End preseason body mass (kg)	93.2 ± 8.1	85.1	86.3	101.0	88.8	104.9
Beginning preseason ƩSF (mm)	85.6 ± 21.2	89.9	64.5	97.8	59.5	116.6
End preseason ƩSF (mm)	75.5 ± 16.4	85.8	57.0	89.0	53.2	90.4
Beginning preseason back squat 1RM (kg)	156.6 ± 9.6	155.0	145.0	150.0	160.0	173.0
End preseason back squat 1RM (kg)	164.6 ± 8.0	160.0	160.0	158	165.0	180.0
Beginning preseason bench press 1RM (kg)	110.5 ± 14.8	102.5	100.0	120.0	95.0	135.0
End preseason bench press 1RM (kg)	115.0 ± 15.5	105.0	105.0	127.5	100.0	140.0
Beginning preseason 30:15 (stage)	19.1 ± 0.5	19.0	20.0	19.0	19.0	18.5
End preseason 30:15 (stage)	19.8 ± 0.5	20.0	20.5	19.5	20.0	19.0

*Kg* kilograms, *mm* millimetres, *Ʃ SK* sum of skinfolds (8 sites), *1RM* one repetition maximum

## Discussion

The aim of the study was to assess the impact of rugby training on markers of gastrointestinal endothelial cell damage and permeability compared to rest. The results show that there was a significant increase in i-FABP levels and lactulose:rhamnose ratio in response to rugby, demonstrating endothelial cell disruption and increased gastrointestinal permeability. These findings are novel in this population and indicate that high intensity intermittent collision-based training will increase gut cell injury, in line with the impact of endurance exercise and high intensity running protocols (Chantler et al. 2020; Pugh et al. 2017a, b). After six weeks of preseason training, there was a similar gastrointestinal cell response in the subsample. Overall, GIS were reported as *none* to *just noticeable* and the markers of initial exercise-associated gut endothelial cell injury did not appear to influence the experience of gastrointestinal symptoms at rest or around rugby.

The significant increase in i-FABP levels matches other studies in endurance athletes (Chantler et al. 2020). In a recent study in marathon runners, there was a significant increase in i-FABP pre to post race of 1129 ± 493 to 2593 ± 1373 ng/l in healthy controls, but levels of 15,389 ± 8547 ng/l were noted post marathon in eight runners who were incapacitated with exercise-associated collapse (Walter et al. 2021). As such, the values reported in this study are not seen as a medical issue, but may be of concern for players in the post-exercise period when nutrition is prioritised as part of recovery (Thomas et al. 2016). This study only evaluated gastrointestinal cell response to exercise, but research from van Wijck (2013) found a correlation between higher plasma i-FABP levels post resistance training and rates of in *vivo* dietary protein absorption, indicating a reduced capacity for the absorption of protein post-exercise with endothelial cell damage (van Wijck et al. 2013a). Contrary, recent research using cow’s milk post-exercise had similar GIS and carbohydrate absorption levels compared to water (measured by hydrogen breath test), in spite of increased i-FABP levels (Costa et al. 2020b). i-FABP levels have been shown to return to normal within 1–2 h post-exercise in healthy athletes, once perfusion is re-established (Schellekens et al. 2017; Pugh et al. 2017a). Therefore, based on limited data, macronutrient absorption may be affected differently by transient endothelial damage, which may be a concern for rugby players in elite settings where energy expenditures are high and positive protein balance is critical (Morehen et al. 2016).

The high intensity rugby session was designed to replicate the physiological demands of match play, although not the duration. The current protocol was ~ 45 min, including warm up, shuttle runs and static collisions (with tackle shields). There was a 70% increase in i-FABP levels in response to the collision-based training. Therefore, a match, with a longer duration (i.e., 80 min), may increase i-FABP levels further. In matches, backs will generally cover more high speed meters and sprints, while forwards will be involved in more collisions (Austin et al. 2011; Reardon et al. 2017). This was not differentiated in this study, but this may be pertinent if future studies consider the role of collisions and positional play. There was some variation in individual i-FABP response, as seen in other studies (March et al. 2017), but the trend was consistent. While the magnitude of change was similar to runners (Pugh et al. 2017b), the absolute values were higher than other studies in endurance sports in temperate conditions (Costa et al. 2020a). This may be due to methodological differences in the analysis, as the players all completed the rugby session after a full rest day and followed similar protocols as previous studies.

The rugby session increased endothelial cell permeability; seen by the significant ~ twofold increase in lactulose:rhamnose ratio. The use of DSAT have been shown to be valid marker of cell permeability (Ogden et al. 2020), as the changes in non-digestible saccharides reflect the increase in tight junction dysregulation and the ability for larger molecules to pass between endothelial cells. The loss of endothelial cell integrity may increase the risk of bacterial translocation across the cell barrier. Increased permeability has been associated with higher levels of inflammatory markers and GIS in response to six weeks of combat training (Li et al. 2013). High intensity exercise has been proposed to have an immunosuppressive effect (Simpson et al. 2020), and any addition aggravation to the immune response via increased bacterial translocation and inflammation may increase the time taken for recovery. While in agreement with the rugby induced change in i-FABP levels, the negative correlation between absolute changes in biomarkers by individual may show that endothelial damage and permeability are not sequentially linked, as has been discussed previously (March et al. 2017). The nature and location of collisions in rugby may alter the mechanical forces on the gastrointestinal system and alter the response of the gut, but this will require further investigation to elucidate the differential impact on biomarkers.

The trend of lower absolute measures of GI permeability after six weeks of training highlights possible gastrointestinal adaptations and would be valuable to repeat in a larger sample and over a longer period. The changes seen in individual players (Table 3) illustrate the positive physical adaptations to the pre-season training program. Pre-season blocks are designed to improve strength outcomes, and aerobic and anaerobic fitness to prepare players for the upcoming season (Argus et al. 2010). A change in relative effort due to improved aerobic fitness, illustrated by the 30–15 IFT, may decrease the level of splanchnic hypoperfusion. Similarly, chronic training may improve the tolerance to training via shifts in microbiome (Bennett et al. 2020); but with the limited sample at follow up, it is difficult to ascertain the full impact of training in this study. Previous data on adaptations to exercise via the microbiome or nutritional exposure supports this as a possible factor (Miall et al. 2018; Keohane et al. 2019). Any improvement in ability to tolerate repeated bouts of exercise for the gastrointestinal lining will be promising for long-term gut health, as hypothetical concerns over recovery time have been highlighted (Van Houten et al. 2015). This may be especially important over the course of a season and direct any future nutrition specific interventions.

Gastrointestinal symptoms were more common at rest than during rugby, but overall severity was low, diminishing concerns over performance related issues in this population. As healthy, well-trained young athletes, this has been shown previously in runners performing repeated 400 m sprints, with no correlation between the mild to moderate GIS and changes in i-FABP (Pugh et al. 2017b). However, considering the profile of NSAID use, travel frequency and high playing volumes in professional rugby, it may be worth establishing if this changes over the course of a rugby player’s career (Van Wijck et al. 2012; Wilson 2018; O’ Donovan et al. 2020).

Unfortunately, the current study did not have an additional control condition without collisions. The addition of physical collisions has been shown to increase the overall energy expenditure and decrease self-reported wellbeing compared to a non-collision training in rugby players (Roe et al. 2017; Costello et al. 2018). It would be useful to explore the role of collision-based activity in isolation in endothelial cell damage and recovery dynamics including nutrient absorption at the level of the gastrointestinal tract. A further limitation includes the small sample at follow up that was unavoidable due to injuries. The study protocol aimed to be more ecologically valid using an on-field design, which makes the results more generalisable to the sport. Formal validation of the testing trial would also be beneficial to strengthen future studies using the protocol.

## Conclusion

This study showed that collison-based rugby training is associated with significant changes in gastrointestinal cell integrity, with no apparent link to GIS. Changes in gut endothelial damage and permeability markers were not proportionate in individuals and may each have a different role in nutrient absorption and recovery that is currently unclear. Chronic training may reduce the impact of the rugby training on gastrointestinal cell integrity, but more research will be needed to better examine any possible gastrointestinal adaptations to training. As there is no data in collision-based sports, this will create an effective starting point for future research around gastrointestinal health in team sport athletes.

## Data Availability

All data in text or in the supplementary material.
